# Biomarkers of Hemodynamic Stress and Aortic Stiffness after STEMI: A Cross-Sectional Analysis

**DOI:** 10.1155/2015/717032

**Published:** 2015-04-16

**Authors:** Sebastian Johannes Reinstadler, Hans-Josef Feistritzer, Gert Klug, Agnes Mayr, Luc Huybrechts, Angelika Hammerer-Lercher, Johannes Mair, Wolfgang-Michael Franz, Bernhard Metzler

**Affiliations:** ^1^University Clinic of Internal Medicine III, Cardiology and Angiology, Medical University of Innsbruck, Anichstraße 35, 6020 Innsbruck, Austria; ^2^Department of Radiology, Medical University of Innsbruck, Anichstraße 35, 6020 Innsbruck, Austria; ^3^Central Institute for Medical and Chemical Laboratory Diagnostics, Medical University of Innsbruck, Anichstraße 35, 6020 Innsbruck, Austria

## Abstract

*Aim*. Increased aortic stiffness might adversely affect cardiac structure, function, and perfusion. Release of biomarkers of hemodynamic stress is thought to be enhanced by these alterations. We aimed to evaluate the association between biomarkers of hemodynamic stress and aortic stiffness assessed at a chronic stage after ST-segment elevation myocardial infarction (STEMI). *Methods*. Fifty-four patients four months after STEMI were enrolled in this cross-sectional, single-center study. N-terminal pro–B-type natriuretic peptide (NT-proBNP), mid-regional pro–A-type natriuretic peptide (MR-proANP), and mid-regional proadrenomedullin (MR-proADM) levels were measured by established assays. Aortic stiffness was assessed by the measurement of pulse wave velocity using phase-contrast cardiovascular magnetic resonance. *Results*. NT-proBNP, MR-proANP, and MR-proADM concentrations were all correlated with aortic stiffness in univariate analysis (*r* = 0.378, *r* = 0.425, and *r* = 0.532; all *P* < 0.005, resp.). In multiple linear regression analysis, NT-proBNP (*β* = 0.316, *P* = 0.005) and MR-proADM (*β* = 0.284, *P* < 0.020) levels were associated with increased aortic stiffness independently of age, blood pressure, and renal function. NT-proBNP was the strongest predictor for high aortic stiffness (area under the curve: 0.82, 95% CI 0.67–0.96). *Conclusion*. At a chronic stage after STEMI, concentrations of biomarkers for hemodynamic stress, especially NT-proBNP, are positively correlated with aortic stiffness. These biomarkers might also be useful as predictors of high aortic stiffness after STEMI.

## 1. Introduction

In the last two decades, multiple studies have shown that increased arterial stiffness is independently associated with cardiovascular morbidity and mortality [1–4]. A recent meta-analysis convincingly confirmed that increased arterial stiffness is a strong predictor of morbidity and mortality in different patient cohorts with cardiovascular diseases [5]. Pathophysiologically, an increase in arterial stiffness is associated with (a) an increase in central pulse pressure, (b) an increase in cardiac afterload, and (c) reduced coronary perfusion due to a decrease in the central diastolic pressure [6, 7]. The current method of choice for the assessment of aortic stiffness is measurement of pulse wave velocity (PWV) [8]. Velocity-encoded, phase-contrast cardiovascular magnetic resonance (CMR) imaging provides a feasible and robust method to assess PWV [9–11].

Natriuretic peptides (NPs), such as N-terminal pro–B-type natriuretic peptide (NT-proBNP) and mid-regional pro–A-type natriuretic peptide (MR-proANP), are synthesised and secreted by cardiomyocytes [12]. Although myocyte stretch is thought to be the main trigger mechanism for the production and secretion of these hormones, other important stimuli might be ventricular hypertrophy, inflammation, ischemia, or fibrosis [12]. Both emerged as important diagnostic and prognostic biomarkers in patients with acute myocardial infarction [13–16]. Mid-regional proadrenomedullin (MR-proADM) is a more stable fragment of adrenomedullin, a vasodilatory hormone, which is primarily secreted by the adrenal medulla [17]. Like natriuretic peptides, MR-proADM is a robust predictor of adverse outcome after acute myocardial infarction [14, 18]. We recently reported an independent association between aortic stiffness measured during the acute phase after ST-elevation myocardial infarction (STEMI) and NT-proBNP levels four months thereafter in 48 patients [19]. In a subgroup of 32 patients comparable correlations were observed for MR-proANP and MR-proADM. The relationship between these biomarkers and aortic stiffness assessed at a chronic stage after STEMI has not been investigated so far. Arterial stiffness might increase myocyte stretch, induce ventricular hypertrophy, and decrease myocardial perfusion, which are all potential trigger mechanisms for biomarker release. Therefore, we measured plasma levels of NT-proBNP, MR-proANP, and MR-proADM and correlated them to aortic stiffness assessed by CMR in a STEMI cohort four months after the index event.

## 2. Materials and Methods

### 2.1. Study Population

From November 2010 to March 2012, 54 eligible patients with first STEMI admitted to University Hospital of Innsbruck were included in this cross-sectional, single-centre study. STEMI was diagnosed according to the redefined ESC/ACC committee criteria [20]. Only patients treated by primary percutaneous coronary intervention within the first 24 hours after symptom onset were enrolled. Patients with a history of a previous myocardial infarction or angiographically proven coronary artery disease, an estimated glomerular filtration rate (eGFR) < 30 mL/min/1.73 m², Killip class > 2 at presentation, or contraindications to CMR analysis were excluded. Patient demographics were assessed by a detailed medical history/examination. The study was approved by the local ethics committee, and written informed consent was obtained from each participant.

### 2.2. Blood Analysis

Heparinized blood samples were collected from all patients 4 months following STEMI by peripheral venipuncture. Samples for NT-proBNP were promptly analysed at the central laboratory of the University Hospital of Innsbruck by personnel blinded to study data. MR-proANP and MR-proADM were measured in batches after storage at −80°C. Assays used for the determination of NT-proBNP, MR-proANP and MR-proADM have previously been described [19, 21]. Briefly, NT-proBNP concentrations were measured using a commercially available assay with an E170 instrument (proBNP II assay using monoclonal antibodies on a Modular, Roche Diagnostics, Vienna, Austria). The analytical limit of detection of NT-proBNP is 5 ng/L and the limit of quantification is 50 ng/L. The intra-assay coefficient of variations (CV) are 1.9% at a concentration of 64 ng/L and 1.2% at a concentration of 2105 ng/L, and the inter-assay CVs are 3.1% at a concentration of 46 ng/L and 2.7% at a concentration of 2170 ng/L according to the package insert. MR-proANP and MR-proADM were measured by fully automated fluorescence immunoassays (Kryptor, Thermo Fisher Scientific, B.R.A.H.M.S., Hennigsdorf, Germany). The analytical limit of detection of MR-proANP is 0.05 nmol/L and the limit of quantification 0.23 nmol/L. The limit of detection for the MR-proADM assay is 2.1 pmol/L and the limit of quantification is 4.5 pmol/L.

### 2.3. Determination of Aortic Stiffness

We used velocity-encoded, phase-contrast CMR imaging for the determination of PWV as described in detail previously [9, 11, 22]. In brief, all scans were performed with a 1.5 Tesla Magnetom Avanto scanner (Siemens, Erlangen, Germany) four months after STEMI. Two slices (128 phases per cardiac cycle) of retrospective ECG-triggered velocity-encoded phase-contrast sequences were set perpendicular to the ascending and abdominal aorta to measure through-plane flow. Spatial resolution was 1.3 × 1.3 × 8 mm. Velocity encoding was set to 150 cm/s and was adjusted in the case of aliasing artefacts. Aortic PWV was calculated as the mean propagation velocity between the ascending and abdominal aorta using the transit time method [11, 23]. Thereby, PWV is defined as the distance between the two aortic levels and the transit time between these sites.

### 2.4. Statistical Analysis

Statistical analysis was performed with SPSS Statistics 19 (IBM, Armonk, NY, USA) as well as MedCalc Version 13.1.2.0 (Ostend, Belgium). Kolmogorov-Smirnov test was applied to test for normal distribution. Results for continuous variables are all expressed as mean ± standard deviation or as median with interquartile range if not normally distributed. Pearson or Spearman-Rho correlations were performed as indicated. To determine whether there is an independent relation between PWV and biomarker levels multiple linear regression analysis was used. Nonnormally distributed variables were log-transformed for multiple regression analysis. Variables with a* P* value < 0.05 in univariate analysis were included into the models. Differences in continuous variables between groups were determined by ANOVA test. To calculate the predictive utility of biomarkers (alone and in combination) for increased PWV, receiver operating characteristic (ROC) analysis was applied. For all data, a two-tailed *P* value of <0.05 was considered to indicate statistical significance.

## 3. Results

**Figure 1:**
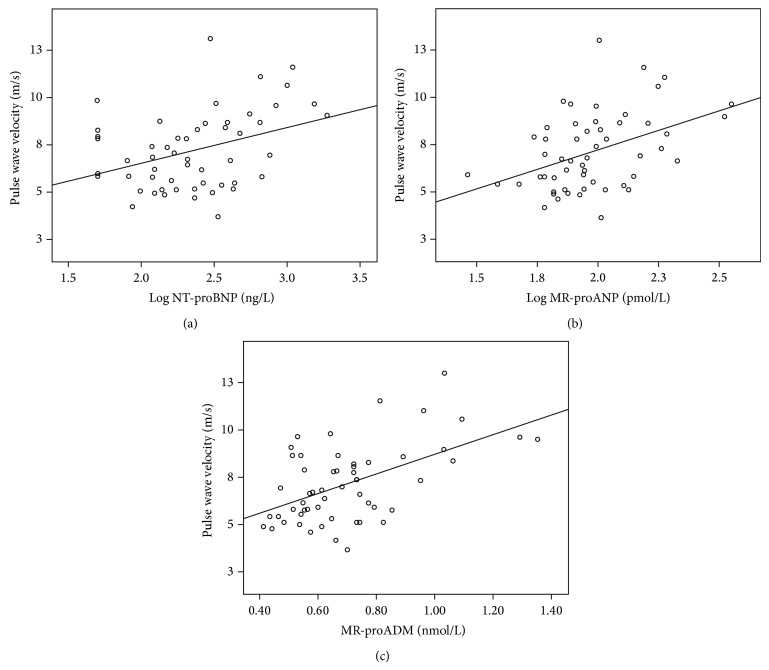
Univariate correlation between plasma NT-proBNP (a), MR-proANP (b), and MR-proADM (c) levels and aortic pulse wave velocity (*r* = 0.378, *r* = 0.425, and *r* = 0.532, resp.; all *P* < 0.005) in patients at a chronic stage after STEMI (*n* = 54).

**Figure 2:**
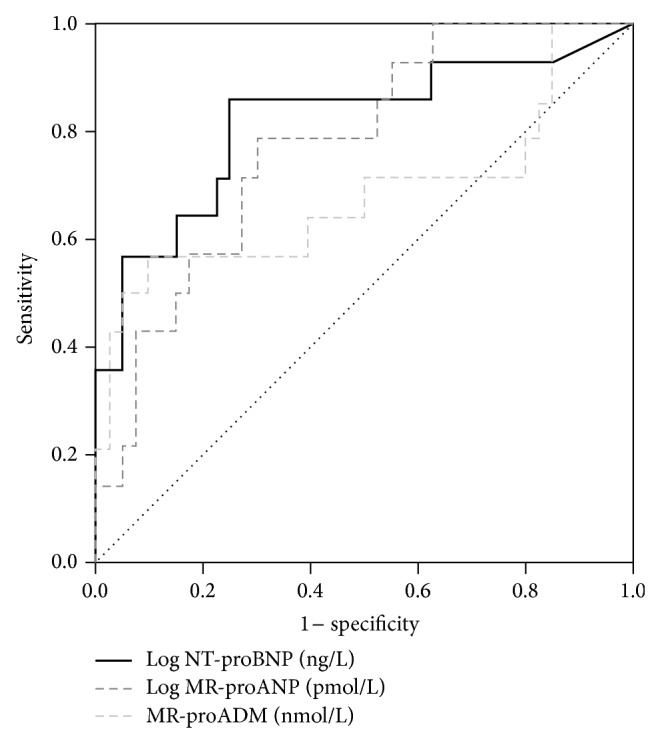
ROC curves for the predictive value of NT-proBNP, MR-proADM, and MR-proANP for increased PWV (=8.6 m/sec, *n* = 14, 26%). The AUCs were as follows: NT-proBNP: 0.82, 95% CI 0.67 to 0.96; MR-proANP: 0.78, 95% CI 0.64 to 0.91; MR-proADM: 0.68, 95% CI 0.49 to 0.88. There was no significant difference between AUCs of each biomarker (all *P* > 0.05).

Table 1 shows the characteristics of the patient cohort. All patients underwent a velocity-encoded, phase-contrast CMR scan for determination of PWV at 129 ± 20 days after STEMI. At that time 54 (100%) patients were on dual antiplatelet- (100% acetylsalicylic acid, 22% clopidogrel, 72% prasugrel, and 6% ticagrelor), 45 (83%) on beta-blocker-, 42 (78%) on angiotensin-converting enzyme inhibitor-, 6 (11%) on angiotensin receptor antagonist-, and 53 (98%) on statin therapy. Mean PWV was 7.2 ± 2.0 m/sec. PWV did not differ significantly between men and women (7.1 ± 1.9 m/sec versus 7.7 ± 2.7 m/sec, *P* = 0.468, resp.). PWV was similar in patients with anterior STEMI and nonanterior STEMI (*P* = 0.547). There was no relationship between PWV and pain-to-balloon time (*r* = 0.046, *P* = 0.740). PWV was strongly correlated to patients' age (*r* = 0.681, *P* < 0.001). No significant correlation was found between PWV and blood pressure, body mass index, total cholesterol, creatinine, and estimated glomerular filtration rate (all *P* > 0.05). There was no significant difference in PWV between patients with or without diabetes (*P* > 0.05). Correlations between PWV and biomarkers of myocardial wall stress are shown in Figure 1. Importantly, log NT-proBNP, log MR-proANP, and MR-proADM were all significantly related to PWV (*r* = 0.378, *r* = 0.425, and *r* = 0.532; all *P* < 0.005, resp.). Partial correlation analysis revealed that NT-proBNP, log MR-proANP, and MR-proADM remained significantly correlated with PWV when adjusting for gender (*r* = 0.367, *r* = 0.415, and *r* = 0.529; all *P* < 0.01, resp.). In multivariate analysis, each marker was examined separately because of the close correlation between them (correlation coefficients between 0.5 and 0.7, *P* < 0.001). In the first model, age, eGFR, systolic blood pressure, diastolic blood pressure, and log NT-proBNP were taken as independent variables. This model revealed that NT-proBNP levels (*β* = 0.316, *P* = 0.005) and age (*β* = 0.627, *P* < 0.001) remained significantly associated with PWV (*R* = 0.758, *P* < 0.001). In the second model, age, eGFR, systolic blood pressure, and log MR-proANP were taken as independent variables. In this model age (*β* = 0.641, *P* < 0.001), but not MR-proANP (*β* = 0.099, *P* = 0.411), correlated with PWV (*R* = 0.709, *P* < 0.001). In the third model, age, eGFR, systolic blood pressure, and MR-proADM were taken as independent variables. Along with age (*β* = 0.566, *P* < 0.001), MR-proADM (*β* = 0.284, *P* < 0.020) remained significantly associated with PWV (*R* = 0.741, *P* < 0.001). Patients were also stratified in those with a PWV below (*n* = 40, 74%) and above (*n* = 14, 26%) the third quartile of PWV (=8.6 m/sec). The area under the curve (AUC) of NT-proBNP (0.82, 95% CI 0.67 to 0.96) with the optimal cut-off level of 270 ng/L revealed 86% sensitivity and 75% specificity for the prediction of an increased PWV. The AUCs of MR-proANP and MR-proADM for the prediction of an increased PWV (MR-proANP: 0.78, 95% CI 0.64 to 0.91; MR-proADM: 0.68, 95% CI 0.49 to 0.88) were lower compared with that of NT-proBNP, but the difference was not significant (NT-proBNP versus MR-proADM: *P* = 0.185; NT-proBNP versus MR-proANP: *P* = 0.525; MR-proADM versus MR-proANP: *P* = 0.284) (Figure 2). The combination of NT-proBNP with MR-proADM (AUC = 0.82, 95% CI 0.69 to 0.91), NT-proANP (AUC = 0.83, 95% CI 0.71 to 0.92), or MR-proADM and NT-proANP (AUC = 0.81, 95% CI 0.68 to 0.91) did not add significant prognostic information (all *P* > 0.300).

## 4. Discussion

In this cross-sectional study of fifty-four patients after first STEMI, we evaluated the association between a 4-month concentration of biomarkers for hemodynamic stress (NT-proBNP, MR-proANP, and MR-proADM) and aortic stiffness. We found significant, positive correlations between these biomarkers and CMR-derived aortic stiffness. Our results suggest that these biomarkers, especially NT-proBNP, might be useful for identifying patients with elevated aortic stiffness as well.

The association between arterial stiffness and cardiovascular risk has been well proven for a long time [5]. Increased aortic stiffness causes hemodynamic and myocardial wall stress, which might stimulate release of NT-proBNP, MR-proANP, and MR-proADM. In fact, an association between arterial stiffness and circulating levels of NT-proBNP has been described in the general population as well as in patients with various diseases [24–28]. This relationship was also observed for patients with stable coronary artery disease (CAD). Şahin et al. showed that in 411 consecutive patients with angiographically proven CAD NT-proBNP levels were independently associated with increased aortic stiffness [29]. Based on their results, the authors speculated that the NT-proBNP value might serve as a predictor of increased aortic stiffness in patients with stable CAD. In contrast, there were only a few studies investigating the correlation between arterial stiffness and MR-proANP or MR-proADM [27, 30, 31]. These studies reported that MR-proANP and MR-proADM are also related to arterial stiffness. Recently, we have shown that aortic stiffness assessed in 48 patients during the acute phase after STEMI is associated with NT-proBNP levels four months thereafter [19]. In a subset of 32 patients, an association between aortic stiffness and MR-proANP as well as MR-proADM was also reported. In the present study, we show for the first time that concentrations of NT-proBNP, MR-proANP, and MR-proADM are significantly associated with increased aortic stiffness at the chronic phase after STEMI. In line with previous studies investigating other populations, the observed correlation coefficients were moderate to good. In ROC analysis, NT-proBNP performed best in predicting increased aortic stiffness defined as the upper quartile of PWV values. Although the sample size of the present study is relatively small, our results indicate that measurement of plasma NT-proBNP concentration at the chronic stage after STEMI could help identify patients with increased aortic stiffness. In this group of patients, assessment of aortic stiffness might be particularly useful for optimizing risk stratification at follow-up. Our results also indicate that aortic stiffening increases the release of NT-proBNP, MR-proANP, and MR-proADM presumably by increasing cardiac afterload also in patients at a chronic phase after STEMI. It is however important to note that there might be other trigger mechanisms than myocyte stretch leading to an increased secretion of this biomarkers in patients after STEMI. Left ventricular hypertrophy as well as fibrosis was shown to enhance gene expression of NPs [32]. Furthermore, stiffening of the aorta leads to an impaired cardiac perfusion [7]. Since myocardial ischemia can also induce production as well as secretion of NPs this might be a further explanation for the observed associations.

Of note, there is enough evidence to show that central arteries stiffen with advancing age [9, 33, 34]. In addition, natriuretic peptides and MR-proADM are also related to age [24, 35]. Our findings confirm these data in patients at a chronic stage after STEMI, since age was closely correlated with aortic stiffness and only moderately with biomarker levels. Other major confounders are blood pressure and renal function [36, 37]. Importantly, in multiple linear regression analysis, plasma levels of NT-proBNP and MR-proADM remained significantly related to aortic stiffness after correction for age, blood pressure, and renal function. By contrast, MR-proANP concentrations were not independently related to PWV in multiple linear regression analysis. A potential explanation for this finding might be that the stimuli for ANP and BNP release might be different, especially in patients with ischemic heart disease [12, 38]. Further studies are necessary to verify this possible explanation.

The cross-sectional design of the study precludes conclusions on a potential causal and temporal relationship between aortic stiffness and the reported biomarkers. Mean PWV was similar to that of mean PWV reported in a previous study assessing PWV in the acute phase after STEMI [19]. Hence, long-term longitudinal investigations with a large number of patients are needed to clarify this question.

A major limitation of this work is the relative small sample size and the fact that females represent only 13% of the study cohort. In partial correlation analysis, the association between biomarkers and PWV remained significant when considering gender. Nevertheless, conclusions regarding gender related differences cannot be drawn from this study. Investigations with a higher patient number as well as a higher percentage of women are necessary to confirm our data and to characterize possible gender differences in detail.

## 5. Conclusions

This is the first study showing that, at a chronic stage after STEMI, levels of NT-proBNP, MR-proANP, and MR-proADM are significantly associated with increased aortic stiffness. Among these biomarkers, especially NT-proBNP might be useful for predicting high aortic stiffness after STEMI. Larger investigations are needed to confirm the results of this study.

## Figures and Tables

**Table 1:** 

Study population (*n* = 54)	
	Mean/median/number
Age, years	59 ± 10
Female, *n* (%)	7 (13)
Body mass index, kg/m^2^	27 ± 3
Family history for AMI, *n* (%)	12 (22)
Smoking status, *n* (%)	25 (46)
Hypertension, *n* (%)	44 (81)
Hyperlipidemia, *n* (%)	36 (67)
Diabetes mellitus, *n* (%)	5 (9)
Pain-to-balloon time, min	261 (129–759)
Anterior STEMI, *n* (%)	17 (32)
Culprit lesion, *n* (%)	
LAD	16 (30)
LCX	10 (18)
RCA	28 (52)
Vessel disease, *n* (%)	
1	24 (44)
2	23 (43)
3	7 (13)
Creatinine, mg/dL	0.98 ± 0.15
eGFR, mL/min/1.73 m^2^	83 ± 15
NT-proBNP, ng/L	219 (119–412)
MR-proANP, pmol/L	88 (68–128)
MR-proADM, nmol/L	0.7 ± 0.2
PWV, m/sec	7.2 ± 2.0

AMI = acute myocardial infarction; STEMI = ST-segment elevation myocardial infarction; LAD = left anterior descending artery; LCX = left circumflex artery; RCA = right coronary artery; eGFR = estimated glomerular filtration rate; NT-proBNP = N-terminal pro–B-type natriuretic peptide; MR-proANP = mid-regional pro–A-type natriuretic peptide; MR-proADM = mid-regional proadrenomedullin; PWV = pulse wave velocity.
